# A Case of Spontaneous Combustion

**Published:** 1847-04

**Authors:** Thomas S. Foster

**Affiliations:** Student of Medicine in the University of Louisville


**Art. IV.—*A Case of Spontaneous Combustion.* Communicated to Prof. Yandell, by Thomas S. Foster, Student of Medicine in the University of Louisville.**

Sir: Your remarks on spontaneous combustion brought to my memory a case which occurred in Shelby county not many weeks since, and which excited much remark in the neigborhood. The particulars were as follows: A negro man seventy years old, who had been a hard drinker for fifty years, was found on his bed, one morning in September, partly consumed by fire. His body was charred, and looked like a lump of coal, while his extremities were untouched, and the bed and bedclothes but little burned. A woman, who slept in an adjoining room, heard no noise in the night, and found him in the condition just described when she entered his room in the morning. If there was a fire in his room at all, it was a small one, as the weather was mild at the time. From all the circumstances attending it, I suppose this may be set down as a case of *spontaneous combustion* in the human subject.

November, 1846.

